# Infrared Spectroscopic Studies of Cells and Tissues: Triple Helix Proteins as a Potential Biomarker for Tumors

**DOI:** 10.1371/journal.pone.0058332

**Published:** 2013-03-20

**Authors:** Allison L. Stelling, Deirdre Toher, Ortrud Uckermann, Jelena Tavkin, Elke Leipnitz, Julia Schweizer, Holger Cramm, Gerald Steiner, Kathrin D. Geiger, Matthias Kirsch

**Affiliations:** 1 Clinical Sensing and Monitoring, Faculty of Medicine, Dresden University of Technology, Dresden, Germany; 2 Department of Engineering Design and Mathematics, Faculty of Environment and Technology, University of the West of England, Bristol, England; 3 Department of Neurosurgery, Faculty of Medicine and University Hospital, Dresden University of Technology, Dresden, Germany; 4 Department of Neuropathology, Institute of Pathology, Faculty of Medicine and University Hospital, Dresden University of Technology, Dresden, Germany; 5 Center for Regenerative Therapies Dresden, Dresden University of Technology, Dresden, Germany; University of Quebect at Trois-Rivieres, Canada

## Abstract

In this work, the infrared (IR) spectra of living neural cells in suspension, native brain tissue, and native brain tumor tissue were investigated. Methods were developed to overcome the strong IR signal of liquid water so that the signal from the cellular biochemicals could be seen. Measurements could be performed during surgeries, within minutes after resection.

Comparison between normal tissue, different cell lineages in suspension, and tumors allowed preliminary assignments of IR bands to be made. The most dramatic difference between tissues and cells was found to be in weaker IR absorbances usually assigned to the triple helix of collagens. Triple helix domains are common in larger structural proteins, and are typically found in the extracellular matrix (ECM) of tissues.

An algorithm to correct offsets and calculate the band heights and positions of these bands was developed, so the variance between identical measurements could be assessed. The initial results indicate the triple helix signal is surprisingly consistent between different individuals, and is altered in tumor tissues. Taken together, these preliminary investigations indicate this triple helix signal may be a reliable biomarker for a tumor-like microenvironment. Thus, this signal has potential to aid in the intra-operational delineation of brain tumor borders.

## Introduction

Vibrational spectroscopy has a long history in analytical chemistry, and many studies rely on these spectra for identifying and analyzing organic molecules [1]. This identification is directly related to the unique frequencies at which particular bonds within the compound vibrate. Certain frequency ranges in a vibrational spectrum may be regarded as unique to specific organic functional groups [1].

The vibrational spectra of tissues contain information about many of the chemical bonds that form the tissue [2]. This information can be used to reliably differentiate normal tissue from diseased tissue [3]. As this discrimination is based upon the biochemical bonds that compose the tissue, these methods have a high potential for providing objective and reliable diagnostic information in a relatively non-invasive manner [3]–[6].

IR studies using both traditional transmission optics and attenuated total reflectance (ATR) optics of human and animal brain tissue date back to the 1950s and 60s [7], [8]. However, most studies using infrared spectroscopy to investigate tissues are performed on dried specimens [7], [8]. This is due to the fact that the IR signal of water is very intense, and can overwhelm signal from the biomolecules [9]. This study endeavored to examine tissues and cells in their hydrated states, to minimize any biochemical changes that might occur upon drying.

IR studies of tissues use small but consistent differences in the absorbance and wavenumber of their spectra to distinguish between different cell lineages [10]–[12], or to monitor their response to drug treatment [13] in a minimally invasive fashion. Most of these studies allowed the cells to air dry so that the strong IR signal of water is removed before the measurements. IR measurements of cells under more native conditions have been performed as well with powerful synchrotron IR optics [14], while others use microscopy to produce chemical maps [15].

In this work, we present the infrared spectra of living cells; and that of native normal and tumor tissues. It was found that using the instrument's anvil to enforce contact with the crystal greatly increased the signal from the biochemicals. A particular spectral region showed interesting alterations between cell, tissue, and tumor spectra. This region is thought to mostly contain vibrations from the unique amide III backbone and proline vibrations from triple helix molecules. These molecules are usually found as large assemblies within the extracellular matrix (ECM) of tissues. We additionally show that after simple spectral pre-processing, the band heights and positions in this region may be reasonably consistent over five normal animals.

The ECM of tissues plays an important role in sustaining tissue cells, and alterations to this microenvironment are thought to play an important role in the transformation of normal tissues into tumor tissues [16]–[18]. This is particularly true for brain tissue, which have a highly unique ECM that is currently thought to regulate neuronal function and growth [19]. Recent work has also shown that certain collagen genes are widely expressed in brain tissues [20]. Tumor cell cultures in particular are known to produce chemically unique ECMs [17].

It is proposed that these bands may represent a unique marker for tumor ECM that relies upon the structurally distinct biomolecules that form this matrix. Additionally, further identification of the biochemical contributions to this band may aid in studies related to non-invasively monitoring cells as they are cultured into tissues. This will contribute to studies seeking to reliably and rapidly distinguish tumor from non-tumor tissue environments using their unique IR signals.

## Results and Conclusions

### The IR of cells versus tissues: methods and preliminary band assignments

A portable IR spectrometer (Bruker Alpha-P, Germany) was used that employs ATR optics to produce a robust instrument that delivers highly reproducible spectra for traditional materials such as solids and dry films. ATR techniques use crystals with a high refractive index, such as diamond, to generate an evanescent wave from an IR source that can penetrate several microns into materials of a lower refractive index [21]–[24]. To achieve the effect, the material under investigation must be placed into extremely close contact with the crystal [22], [23].

**Figure 1:**
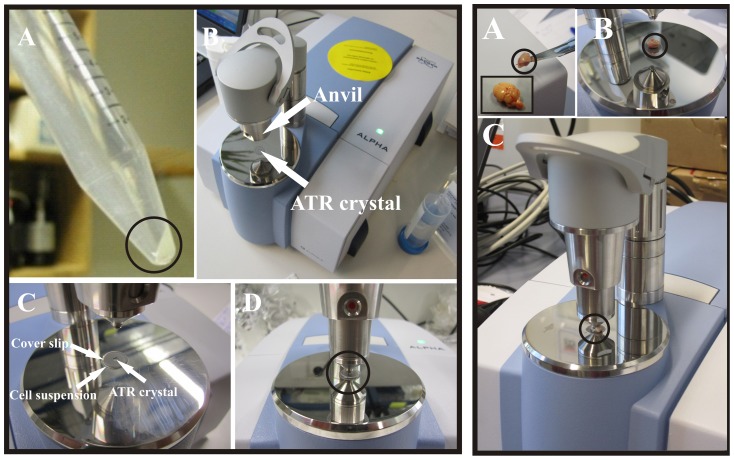
Methodology for ATR infrared measurements of cells and tissues. Left: (A) Picture of a typical cell suspension (circled). (B) The portable ATR FT-IR spectromenter by Bruker. An arrow is pointing to the ATR diamond crystal. For measurements, a 10 µl aliquot of the cell suspension is pipetted on to the crystal. (C) A CaF_2_ coverslip is placed on top of the 10 µl droplet, and (D) the anvil of the instrument is lowered so that the cell suspension will achieve good contact with the crystal optics. Right: (A) Shown in the boxed inset is a freshly extracted mouse brain, with a typically sized mouse brain piece on the scalpel (circled). After the brain was cut into small pieces, one was (B) placed on the ART crystal, and (C) the anvil was lowered and the ATR spectrum taken. While spectra could be obtained from tissue without lowering the anvil, the resulting spectra had inadequate signal to noise (data not shown). Pathology results (see figure 2C) indicated that the damage sustained by the tissue piece from the anvil is minimal, if only one or two spectra are taken with the anvil down.

**Figure 2:**
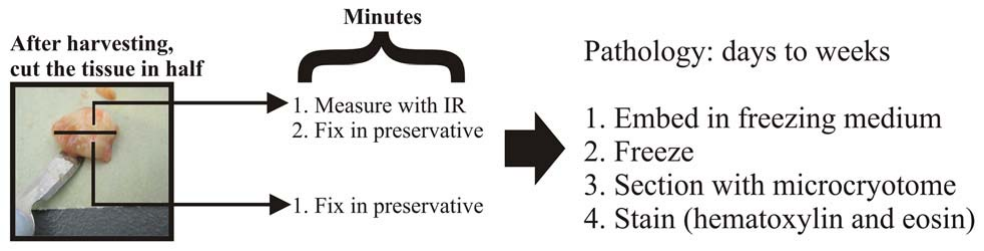
Flowchart for tissue IR experiments. Immediately after the tissue is harvested, the tissue is cut in halve. Once half is immediately fixed in a chemical preservative, and the other fixed after it is measured with IR. The pathology results for the unmeasured control and the measured tissue were then obtained to arrive a diagnosis.

Figure 1 shows the methodology used to obtain infrared spectra of cells (left) and tissues (right). Figure 2 shows a flowchart for the tissue measurements. It was found that compressing the cell suspension against the ATR crystal with a CaF_2_ cover slip (see figure 1) greatly enhanced the IR signal from the cells over the strong absorbance of liquid water, see figure 2. This is in line with the ATR optical effect, as the evanescent wave has an extremely short (microns) penetration depth into the material placed on its surface [23]. In the cell suspension measurements, the use of the CaF_2_ cover slip likely forces an exact beam path by evenly spreading the liquid over the crystal. This allows comparisons in band heights to be made between different measurements without the need for normalization.

**Figure 3:**
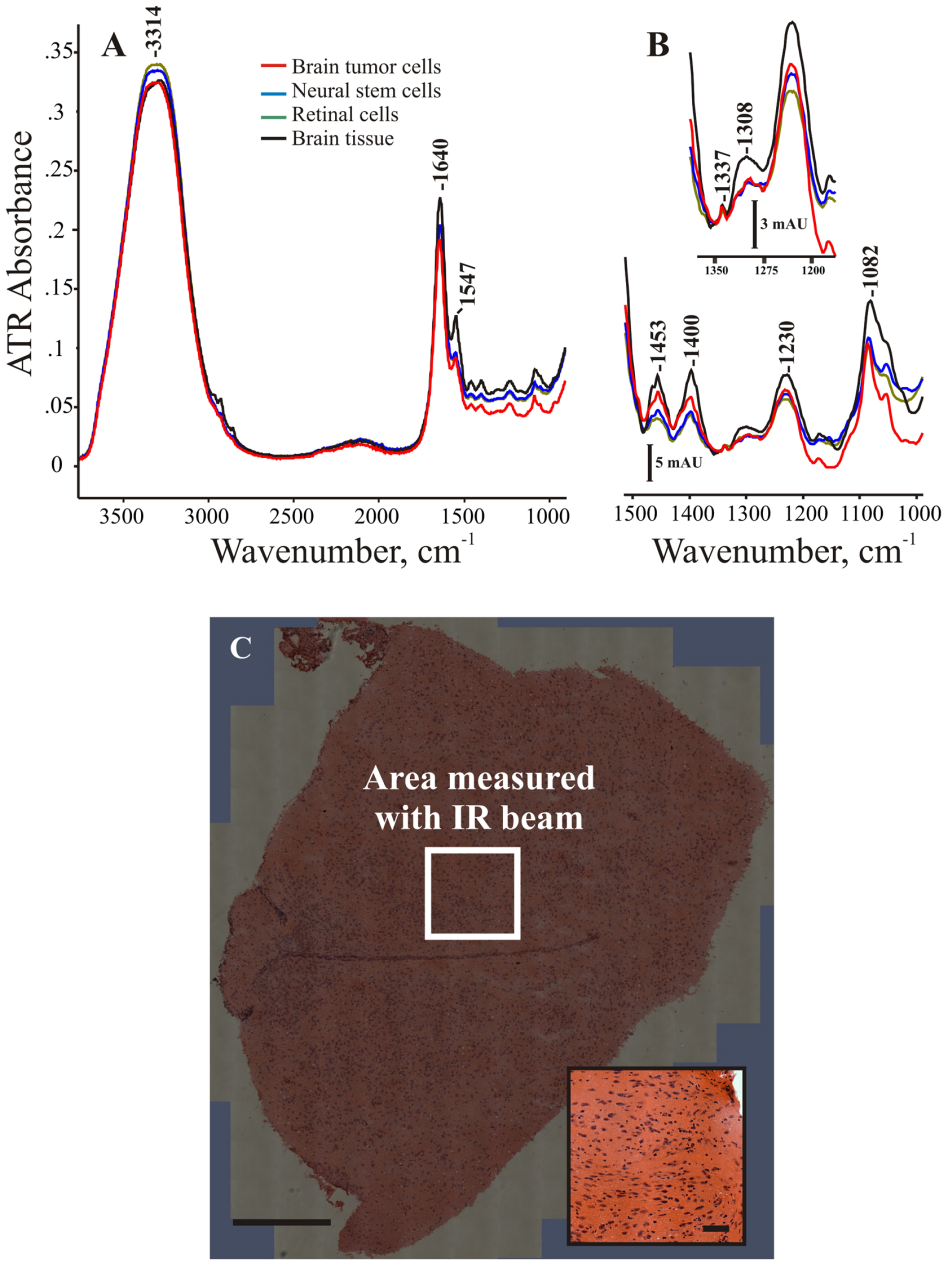
ATR spectra of cells and tissues. (A) The unprocessed, raw infrared spectra of wet cells and tissues are shown. Most of the IR signal is due to water vibrations at 3313 and 1640 cm^−1^. (B) Enhanced view of the biologically relevant fingerprint region. Absorbencies in this region arise from biochemical bonds that form cells and tissues. The spectra of cells are quite similar to the spectra of tissues, expect in the triple helix region (inset). (C) Hematoxylin and eosin stain for an unmeasured piece of normal mouse brain tissue adjacent to the measured piece. The white box approximately indicates the 0.25 mm^2^ area of the tissue interrogated with the infrared beam for this ATR instrument. The insets shows an enhanced view from the tissue section. Scale bars: overview, 500 µ; inset, 50 µ.

Figure 3A shows the full IR spectra from brain tumor cells, neural stem cells, retinal cells, and normal brain tissue. The signal of liquid water is quite intense in the unprocessed spectrum, with a height of 0.35 ATR absorbance units (AUs) (about 1.2 AUs after an ATR correction algorithm is applied). This strong water signal overwhelms the signal from the cellular biomolecules, which usually fall between 1800 and 900 cm^−1^, if the anvil of the instrument is not used (data not shown). As tissues are more dense than cells floating in solution, their IR signal is slightly more intense (figure 2B and inset).

Figure 3C shows the hematoxylin and eosin stain for a section of tissue adjacent to a piece that was measured. The white box shows the approximate area the ATR beam is measuring, which only illuminates the first several microns of the tissue. The high similarity in both the signal intensity and band positions between cell and tissue spectra (see figure 3A and B) indicates that the observed bands contain contributions from most of the biochemicals present in a 0.5 by 0.5 mm area. This similarity is likely due to the fact that neural, retinal, and even tumor cells in suspension and normal brain tissue are composed of similar biochemicals. These results indicate this particular IR measurement is insensitive to anything but dramatic chemical alterations in hydrated materials and liquid water.

As can be seen from figure 3A and B, there are reasonably intense bands at 1453, 1400, 1230, and 1082 cm^−1^. This is in line with many previous vibrational studies on both tissues and cells, which have assigned these to various biochemical functional groups [3], [4], [15], [25]. The intense bands at 1640 and 1547 cm^−1^ contain contributions from liquid water [9]. Although these bands do contain vibrations from the amide backbone in proteins and are intense in dried tissues [3], [4], [15], [25], the strong signal of liquid water makes interpretation of these signals difficult.

In fact, the only changes that can be seen in the raw, unprocessed spectra are for weaker bands that occur between 1200 and 1380 cm^−1^ (see figure 3B, inset). In mouse tissue, a broad band is seen centered at 1308 cm^−1^. In the spectra of cells, this band is weaker than tissues and appears to broaden, with shoulders at 1280, 1300, and 1320 cm^−1^ (see figure 3B, inset and table 1). This region of the vibrational spectrum is usually assigned to the amide III vibration of proteins and CH_2_ wagging modes [26]–[28]. These particular frequencies are usually attributed to the left handed triple helices from which structural extracellular matrix proteins like collagens are composed [26]–[28].

**Table 1:** Collagen-like infrared bands in wet cells and tissues [26]–[28].

Assignment	Cells	Tissue	Meningioma	Collagen Film
ECM	1299(br)	1308(br)	-	-
Amide III in Triple Helix	-	-	1238	1240
Amide III in Triple Helix	1280 (w)	-	1280	1280
Proline CH_2_ in Triple Helix	1315(w sh)	-	1318	1318(sh)
Proline CH_2_ in Triple Helix	1337(w)	1337(w)	1337	1337

Table of IR bands for the triple helix region of neural cells and brain tissues. Units in wavenumber. W, weak; sh, shoulder; br, broad.

**Figure 4:**
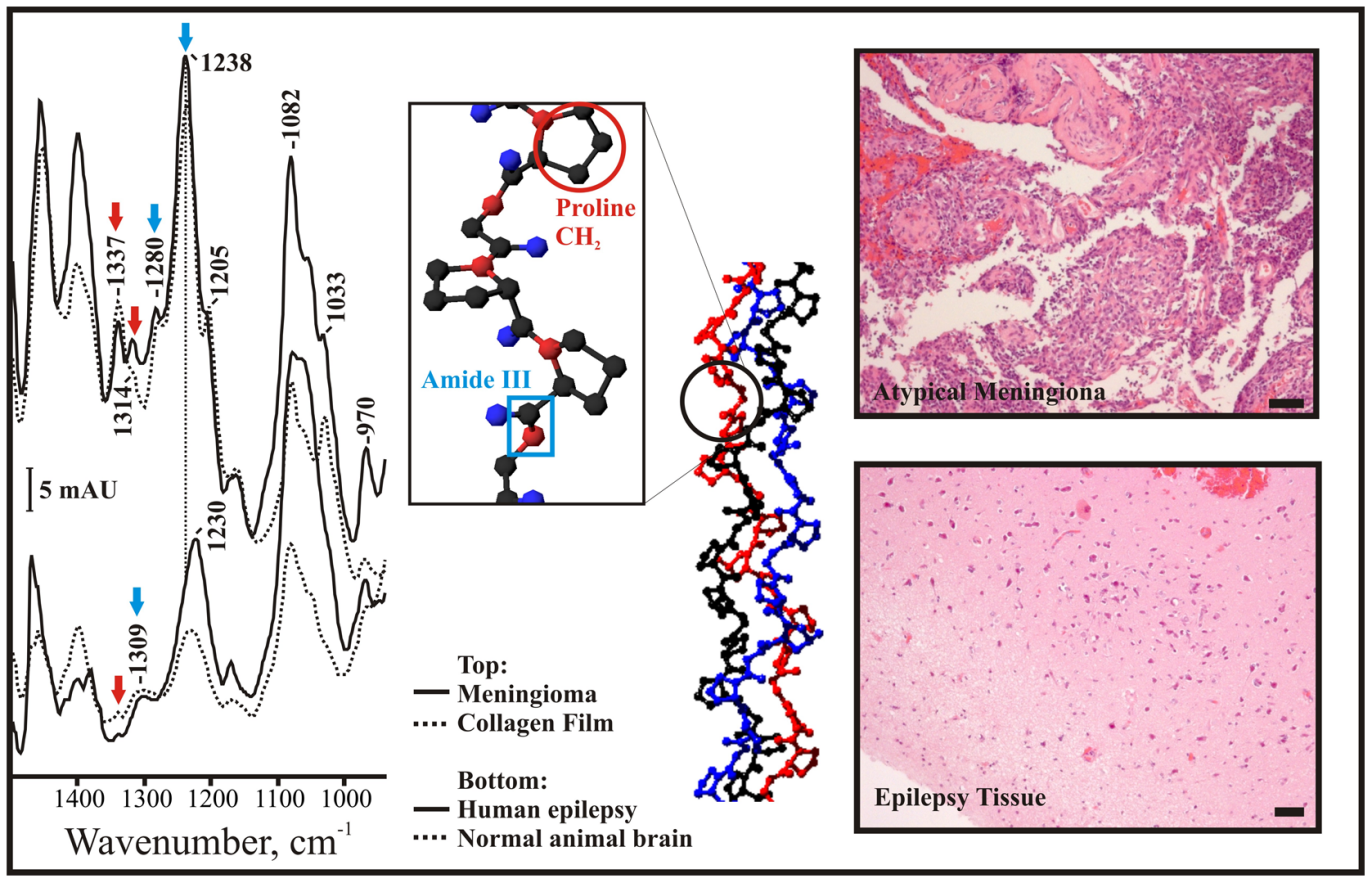
ATR spectra of normal and tumor tissues. Left: Infrared spectra of normal animal brain tissue (bottom, dashed), human epilepsy brain tissue (bottom, solid), an atypical meningioma WHO II (top, solid), and a thin film of collagen (top, dashed). Blue arrows indicate the amide III vibration of the collagen triple helix, while red arrows indicate vibrations from the proline CH_2_ groups that are abundant in these molecules. Middle: Crystal structure of a model collagen showing the triple helix structure. A proline reside is circled in red, and a C-N amide bond boxed in blue. From: Okuyama et al. (Biopolymers, 2009 91:361; PDB ID: 2D3F [42]). Right: The top shows the pathology results for the atypical meningioma spectra, while the bottom shows the pathology for a patient with epilepsy. Scale bars: 50 µ.

Shown on the bottom right in figure 4 are infrared spectra from a mouse (bottom, dashed) and a patient with epilepsy (bottom, solid). In the middle of figure 4, a model collagen triple helix peptide is shown. The amide bond is boxed in blue, and the proline CH_2_ groups are circled in red. The human and mouse spectra are remarkably similar to each other in the triple helix region (see arrows), with a broad band at 1308 cm^−1^.

This similarity is also seen in their pathology. The mouse (figure 3C) and the human epilepsy (figure 4 right bottom) sections look fairly similar to each other, with an even distribution of small nuclei (dark purple) throughout the extracellular matrix and cytoplasmic protein components (light pink).

On the top right of figure 4, the spectra from a patient with an atypical meningioma is shown (solid). A spectrum from a collagen film is overlaid for comparison (dashed). Both spectra show fairly strong IR signals at 1337, 1314, 1280, and 1240 cm^−1^. Meningiomas are known for having high amounts of collagens in their ECMs [29], and this appears to be reflected in their infrared spectrum. The signal at 1337 cm^−1^ is usually attributed to the CH_2_ vibrations from prolines within a triple helix (red arrows) [26]–[28]. The 1280 and 1240 cm^−1^ bands (blue arrows) are usually assigned to the amide III vibrations from the peptide backbone of a triple helix (see blue box) [26]–[28].

Although reasonably strong in the collagen film and the meningioma, these signals are quite weak in normal tissues (see figure 4, bottom). As normal brain tissue does not express as many ECM structural molecules as tumor tissue appears to, the band at 1337 cm^−1^ is either very weak or absent. Normal hydrated tissues appear to have a weak, broad band at 1309 cm^−1^ and a stronger absorbance at 1230 cm^−1^ (figure 4, bottom). This is in contrast to the distinct bands seen at 1337, 1280, and 1240 cm^−1^ in the meningioma and collagen spectra (figure 4, top).

The larger absorbance in the tumor spectra indicates that the meningiomas may have more triple helix proteins per 0.25 mm^2^ of tissue than normal does. Preliminary experiments over multiple animals and two different cell lineages were performed, to examine the variance of the IR signal between identical measurements.

### The heights of tissue amide III bands are reasonably consistent over identical measurements

**Figure 5:**
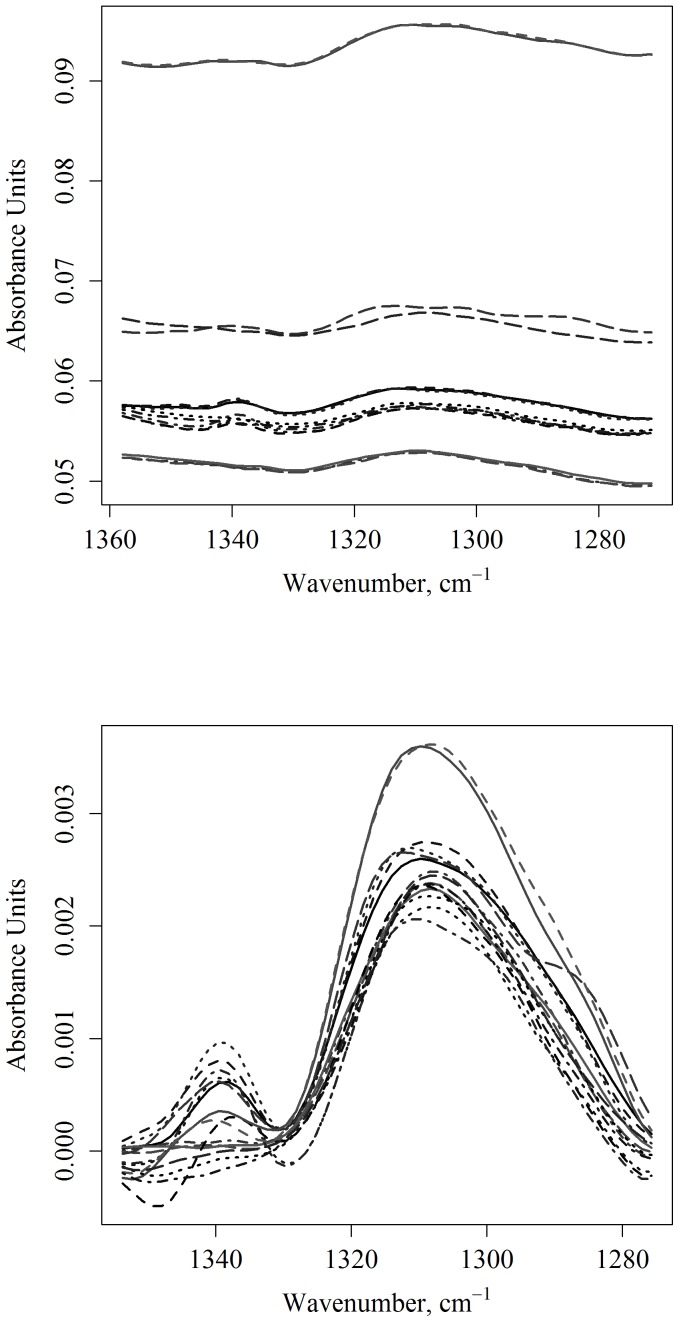
Spectral preprocessing and band height analysis. On top is shown the unprocessed region from 1356 to 1273 cm^−1^ for the animal spectra. It was possible to obtain three spectra from each animal brain tissue before effects due to water loss were seen in the IR. On the bottom is shown the now corrected spectra for five mice. After correcting for the offset and baseline, greater agreement is seen between the spectra of different animals.

**Figure 6:**
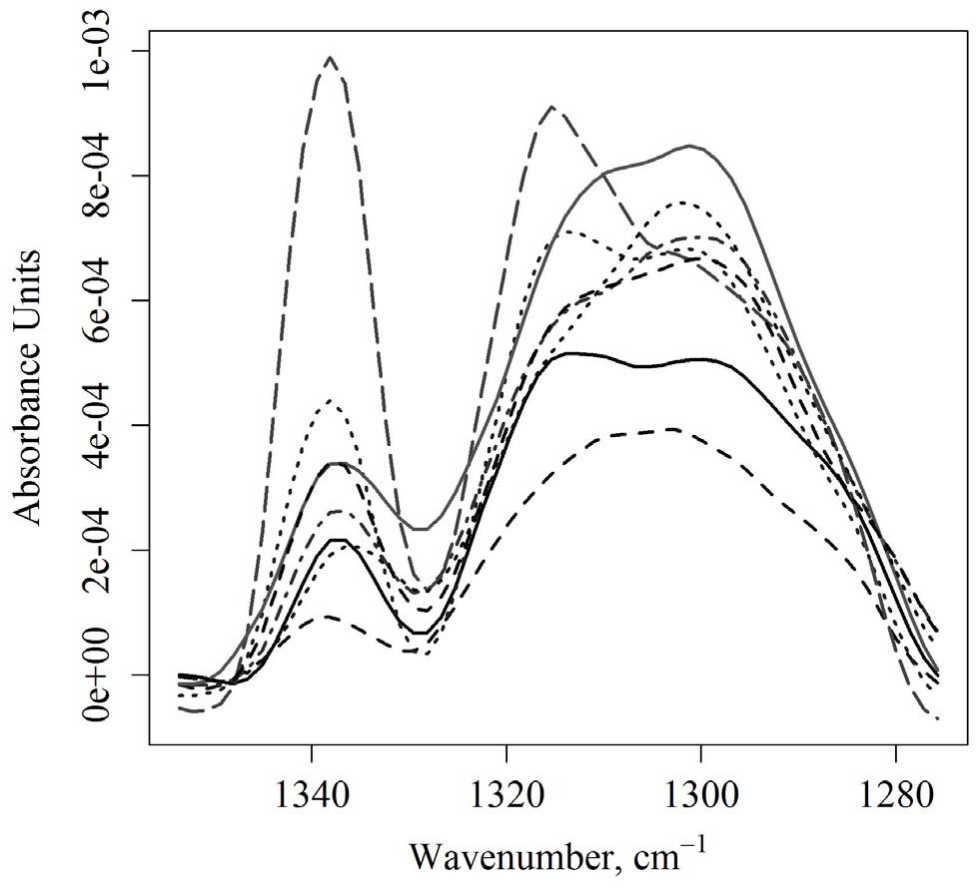
Corrected triple helix region in a retinal cell culture. The R28 rat retinal cells show a broad, weak collagen band whose height is consistent for the same cell line over multiple passages. All spectra were collected on the same day over four different passages. Two spectra could be taken per passage.

**Figure 7:**
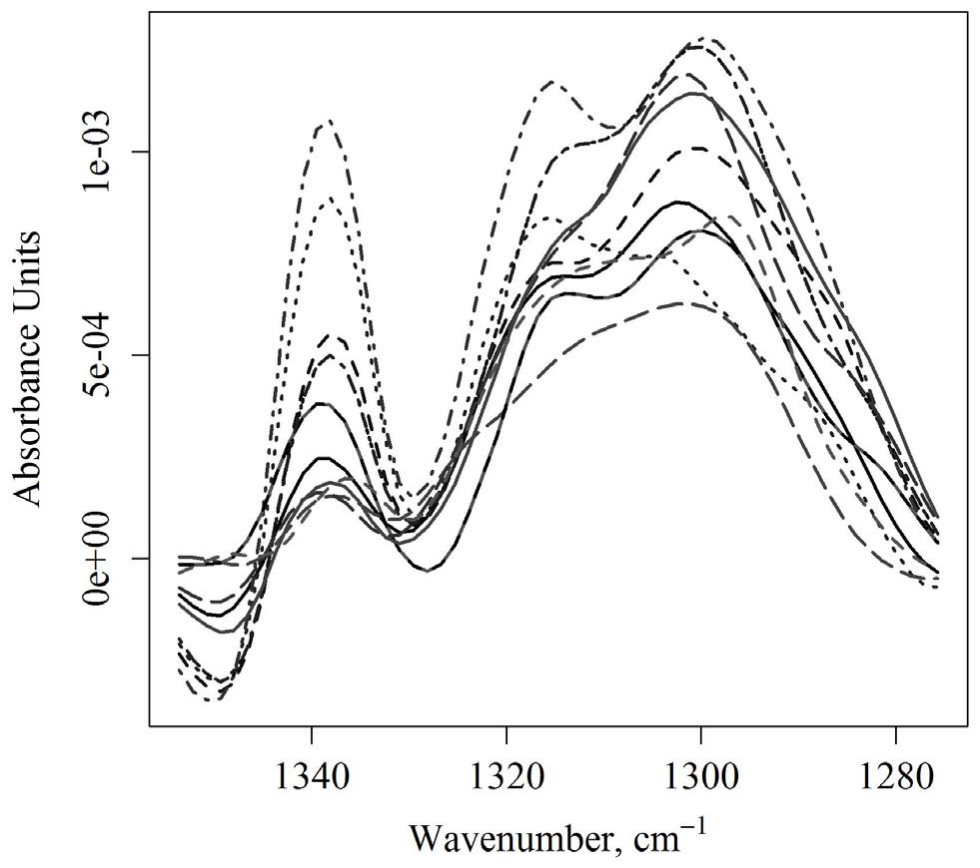
Corrected triple helix region in a human tumor cell culture. Fast growing human glioblastoma cells. These spectra were taken over the course of three days, with two to five measurement per cell suspension.

Figure 5 shows the corrected amide III spectral region of the tissue spectra. Shown on the top of figure 5 are the uncorrected spectra for five animals, with three measurements per animal. The bottom plot shows the spectra for these animals after the correction is applied. Figures 6 and 7 show the corrected spectra spectra in the amide III region from identically prepared cell suspensions. The spectra of the cells in suspension have higher variance than the tissue spectra, although all are surprisingly consistent given the sensitively of IR measurement to environmental errors and the low absorbance of the band.

**Figure 8:**
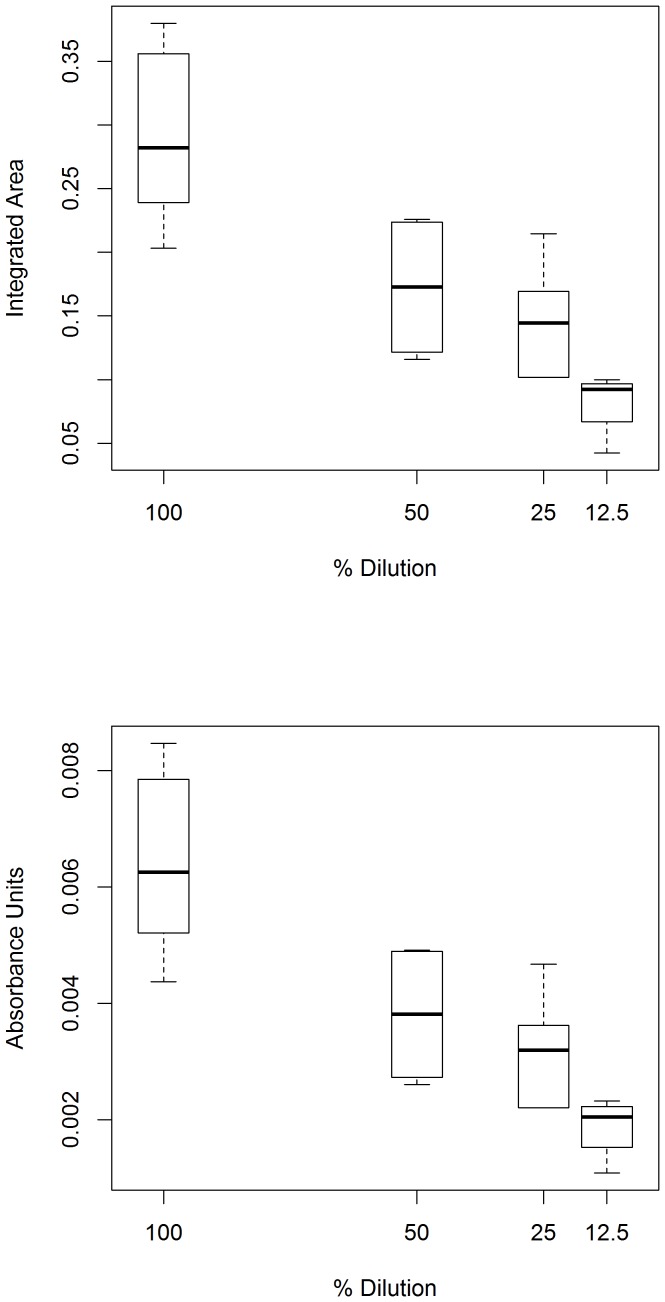
The ATR IR signal decreases with decreasing dilutions of cells. Shown is a box plot for serial dilutions of a glioblastoma cell suspension. As the dilution number decreases, both the area of the 1230 cm^−1^ band (top) and its height (bottom) appear to decrease as well. This indicates that the ATR IR signal from the cells might depend upon the density of cellular material within the beam path of the instrument. It may be the case that compression with the instrument anvil allows the beam path to be set, reducing variability in absorbance between identical measurements. The number of measurements for the 100% dilution was 12 spectra, for the 50% and 25% dilutions six spectra, and for the 12.5% dilution was four spectra. Measurements were performed over the course of three days.

To demonstrate that the IR signal is sensitive to the amount of cellular material present within the ATR beam path, a series of dilutions were made from several identically prepared tumor cell suspensions, see figure 8. For these plots the minima of the 1230 cm^−1^ band were set to zero AUs, after setting their absorbance values equal to each other at each end point. The 1230 cm^−1^ band was used for this analysis as the amide III band is too weak to analyze upon dilution. The values for the integrated area under the band (top) and the band height calculated from the band maxima (bottom) are plotted in figure 8.

There is an approximately linear relationship between the ATR IR signal and the level of dilution for these fast growing cells, see figure 8. The compression of the anvil (see figure 1) appears to fix the beam path of the ATR wave to a certain degree for liquid materials. The decrease in absorbance with the percent dilution (figure 8) also indicates that normalization procedures do not need to be applied to compare the band heights between different measurements and samples. The ATR infrared signal varies with the number of cells in the beam path, indicating that there are few non-linear effects taking place that could alter the band heights.

Additionally, the variation of the values for both integral absorbance and band height for the 1230 cm^−1^ band at 100% dilution are quite high (see figure 8). This indicates that the stronger bands in the IR spectrum of hydrated tissue may be affected by hydration content alone. These bands are not suitable for analysis, as there is too much variance in their values between identical measurements performed on water-laden materials.

These results indicate this methodology (figure 1) results in spectra with reasonably consistent band heights between identical animals and cell suspensions after simple corrections are applied for the triple helix spectral region (see figures 5, 6, and 7).

The agreement between the different individual animal spectra shown in figure 5 appears reasonably consistent. The band height value across five different animals was found to be 0.0026±0.00043 AUs with a maximum at 1308 cm^−1^ (see table 2). Table S1 in File S1 shows the averaged values for the individual mice across three measurements. The individual values for the calculated heights and positions of the proline and amide III bands can be seen in table S2 in file S1.

**Table 2:** Band heights, positions, and deviations for rat retinal cells, human brain tumor cells, and normal mouse brain tissue.

Sample	N	Band height ± standard deviation (AUs)	Position(s), cm^−1^
retinal cells	8	0.00069±0.00016	1300, 1315(sh)
tumor cells	12	0.001±0.00022	1300, 1315(sh)
brain tissue	5	0.0026±0.00043	1308

The heights and positions of the ECM bands are reasonably consistent over a range of biologically identical prepared samples. The retinal cells from the R28 cell lineage were all measured on the same day, but from different passages of the genetically identical cell line. Two measurements were performed per passage. The tumor cells were from a human glioblastoma cell culture. Measurements for these rapidly proliferating cells were performed over three days. For the cell suspensions, the average and standard deviation was calculated with all values treated as independent, identical measurements. The mouse spectra were collected from five animals over the course of two days. Three measurements were performed for each mouse. Shown is mean of means and standard deviation for the five animals. These results indicate this less intense amide III signal is above the signal to noise for the measurement. A larger number of N is required to determine if identical experiments produce values that are not statistically different from each other. Sh, shoulder.

In normal tissue, the proline band at 1340 cm^−1^ is quite small or not present, and this is reflected in the minuscule band heights (see table S2 in file S1 for the calculated values from the animal tissue). However, the amide III signal does appear to be above the noise for tissues with a reasonable standard deviation across five individuals; see table 2 for the calculated mean of means and table S1 in file S1 for the raw values from individual mice. Further trials over a higher number of animals are required to confirm whether this variation has merely occurred by random chance or if there is a systematic variation present of the amide III signal within individuals.

Shown in figure 6 is the corrected amide III region for rat retinal cells in suspension across four different passages. Two measurements could be taken for each passage. As cells in suspension are not as dense as tissue, the IR signal from cells is much lower in absorbance. While this number of repetitions is too low for statistical analysis, it can be seen from their corrected spectra in figure 6 that these genetically identical cell cultures exhibit a goodly degree of variation in the amide III band. Indeed, the average value for the amide III signal is 0.00069±0.00016 AUs centered at 1300 cm^−1^ (see table 2), much lower than the tissue signal and with a higher variance.

This variance seen in genetically identical passages could in part be due to slightly different amounts of collagen-like ECM molecules that could exist between identically prepared cell suspensions; although the low number of experiments could also account for the variance. Table 2 shows the averaged value for their band heights across eight measurements, and table S1 in file S1 shows the averaged values for from four separate passages with two measurements per passage.

Shown in figure 7 are human glioblastoma cells in suspension. As with the rat retinal cells, the tumor cells are much lower in absorbance than the tissue cells. However, both cell lineages (figures 6 and 7) do appear to have altered band positions than seen in tissue for this ECM region. Namely, the amide III band centered at 1308 cm^−1^ in brain tissue appears to be splitting into a broad band with shoulders at 1320 and 1280 cm^−1^. Further purification studies of different cell lineages in suspension would aid in the molecular interpretation of the tumor spectra. Such studies would also determine the amount of variance that exists between measurements on identically prepared cell suspensions.

In conclusion, although weak, the amide III band is above the signal to noise for this instrument in both cell suspensions and tissues. It appears to vary less between genetically identical individuals than the stronger signals do under hydrated conditions. This suggests that the amide III region is more molecularly specific than the stronger IR bands seen in cells and tissues (see figure 2B), and less likely to vary between identical experiments.

In contrast, a similar analysis of the stronger bands at 1230 cm^−1^ reveals that they have a high variance between identical measurements and different individuals (see the error bars in the 100% dilutions in figure 8). This indicates the stronger bands are likely composed of a high number of absorbencies from different phosphates, sugars, and nucleic acids; especially under native, fully hydrated conditions. The high number of contributions broadens these strong bands and makes them less specific on a molecular level. They appear more sensitive to chemical and environmental differences that exist between genetically identical animals and cells. Thus, these stronger bands are more variable than the weaker, but more specific, triple helix bands. Therefore, in the tumor tissue spectra, only the triple helix region of the spectra was investigated.

### Infrared spectra of freshly harvested meningiomas and glioblastomas

Recent studies on the diffusion of fluorescent dyes and other small molecules into freshly resected human glioblastoma tissue indicate that these tumors may have a particularly dense ECM [30], [31]. Many tumor pathologies involve the overproduction of ECM genes for the secretion of large, structural proteins that assemble in the extracellular space [17]. Triple helix domains are found in many of these secreted proteins and protein assemblies [17]. These assemblies may combine in various ways to confer a range of different micro- and macromolecular properties to the tissue; such as hardness, viscosity, and conductivity. Meningiomas in particular are known for their dense and complex ECMs, and the spectrum shown in figure 1 has particularly strong triple helix bands in this individual (see arrows).

As mentioned, the top right of figure 4 shows a spectrum of freshly harvested meningioma tissue. The triple helix signal is strong, though not as strong in all studied to date. Tables S3 and S4 in file S1 list the band positions and heights for more patients for the triple helix signal. The proline band increases in height in some cases by an order of magnitude, and the amide III appears to increase and alter its position in most cases to date.

**Figure 9:**
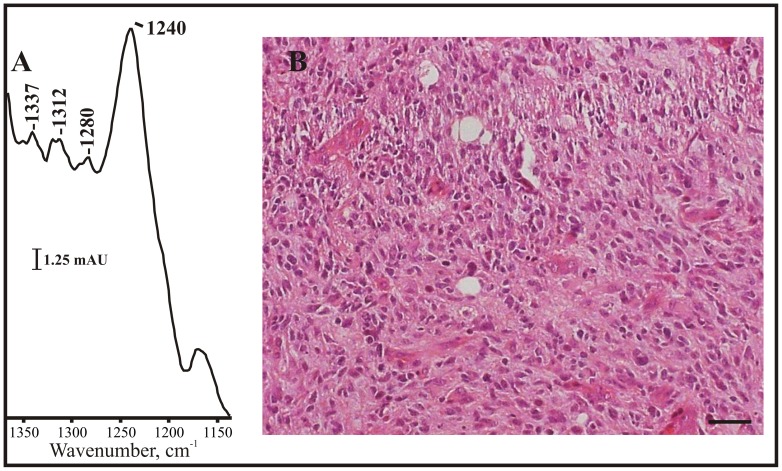
The triple helix signal in the IR spectrum of glioblastoma tissue. (A) The triple helix region of the infrared spectrum from a patient with a glioblastoma. The triple helix signal is weaker than seen in the meningiomas, which could be due to differences in the expression of these matrix proteins. (B) Hematoxylin and eosin staining of the tissue. Scale bar: 25 µ.

Figure 9A shows an uncorrected spectrum from a patient with WHO grade IV glioblastoma. The pathology is shown in 9B. In glioblastoma cases, the proline bands at 1337 cm^−1^ and 1320 cm^−1^ are more pronounced than in the spectrum of normal tissue, and the amide III starts to develop a shoulder at 1280 cm^−1^ (see tables S3 and S4 in file S1). The 1240 cm^−1^ amide III band is slightly red shifted towards the value seen in normal tissue (1230 cm^−1^, see figures 2 and 9), though not in all cases.

**Figure 10:**
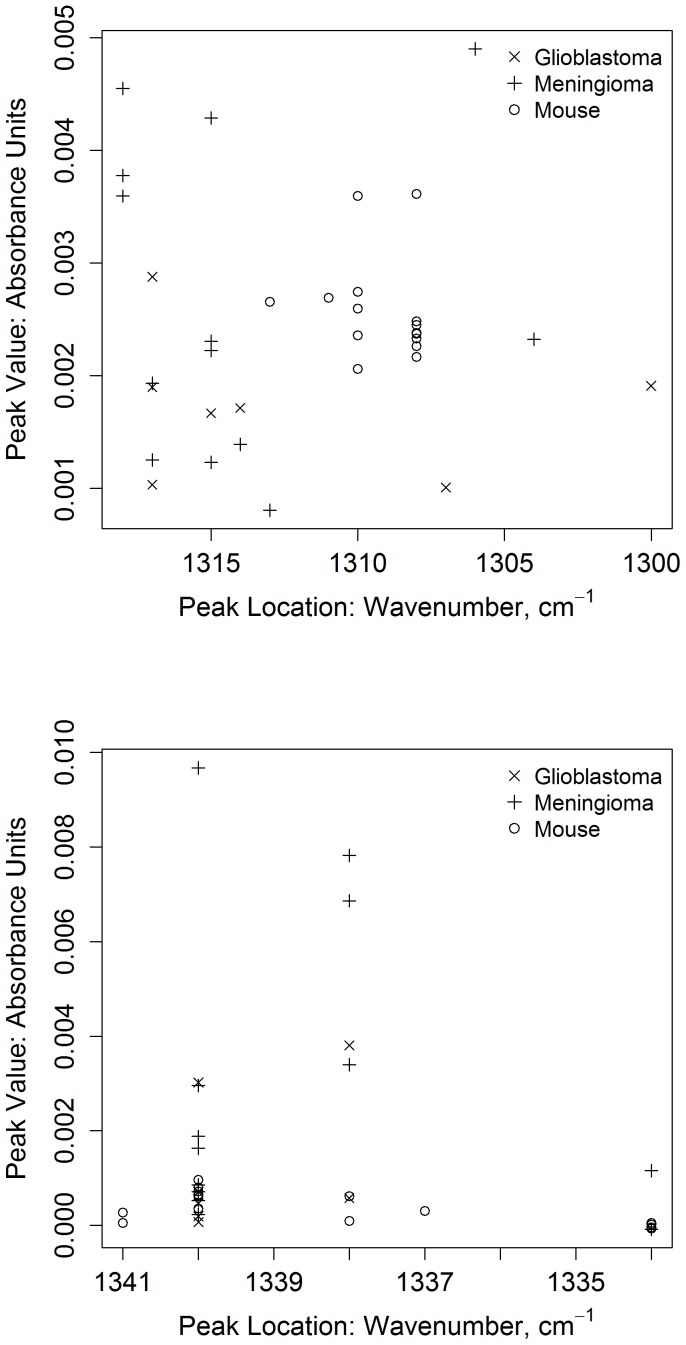
Plot of the band heights and positions for meningiomas, glioblastomas, and normal tissue. The band height is plotted against the band position for the amide III band (top) and the proline band (bottom) for meningiomas (+, n = 13), glioblastomas (x, n = 7), and normal mouse tissue (o, n = 5 individuals, 3 measurements per individual). One spectrum was taken for the tumor measurements, and three spectra were taken for each mouse. The amide III values appear to have less variance than the proline values for normal tissue. The amide III values (top) for most of the tumors appear to be different from those seem in normal tissue. The proline values (bottom) for many meningiomas are quite high compared to the glioblastoma and the normal tissue values.

Figure 10 shows a plot of amide III (top) and proline (bottom) values from meningiomas and glioblastomas. Values from the 15 spectra taken from normal mouse tissue are plotted for comparison. The variance for glioblastomas appears higher than the meningiomas for these numbers, see tables S3 and S4 in file S1. Both tumor pathologies appear to be distinct from the values seen in the normal tissue. The band positions of the tumors in particular shift quite a bit from the values seen in normal tissue, indicating a basic change has occurred in the biochemicals they express.

The glioblastoma values vary quite a bit between individuals, as expected (figure 10). Glioblastomas are known for displaying a high amount of inter-individual variance, making their diagnosis and treatment difficult [32], [33]. This is reflected in the widely varying values for these patients seen thus far, though most appear to be different from those seen in normal tissue (see figure 10). Recent studies have also shown that extracellular collagen genes are differentially regulated in patients with glioblastomas, although there are differences in the expression patterns between individuals [34]. This appears to be reflected in their IR spectra. The values thus far are different from normal tissue, but show a large amount of variance within the pathology (see figure 10). This indicates that the glioblastomas, as expected [34], express highly unique structural proteins that exhibit a large amount of inter-individual variance.

In conclusion, the triple helix bands in the glioblastomas (figure 9) are usually more distinct than seen in the normal mouse tissue (figure 2), but do not seem as enhanced as the ones seen in meningioma patients (see figure 4 top right and tables S3 and S4 in file S1). This indicates that this ATR infrared measurement could be sensitive to the amount of triple helix molecules in the tumor tissue. Thus far this appears to be strongest in the meningiomas, weaker in glioblastomas, and exists as a broad absorbance in normal tissues.

### Conclusions

Taken together, these results from cells, tissues, and tumor patients demonstrate the triple helix bands represent a potential biomarker for tumor tissues. As no sample preparation is required and the methodology is minimally invasive, investigated tissues could be stored for further examination with traditional pathology. Due to the portability of the instrument, spectra from patients were obtained within minutes after resection.

Collagen is the most abundant protein in the body. However, most studies of brain tissues to date are focused on genetic and morphological analysis of the cells. Recent research is starting to untangle the underlying biomolecules that grants brain tissue its unique physical properties [17].

Characterization of brain tissue typically involves embedding the tissue in a freezing medium, freezing the tissue, and cutting it into thin sections. After the tissue is sectioned, it can be stained with a range of dyes to enhance morphological contrast for microscopy. During these procedures, it may be that much of the matrix scaffolding molecules are lost. It is already known from FTIR microscope imaging studies that the IR signal of collagen can be seen in tumor tissue sections after freezing and air drying [35]. More triple helix domains would be expected to be present in the freshly harvested tissues and living cell suspensions used in this study.

This work demonstrates that the IR signal of collagen-like triple helix proteins can be seen in freshly resected tissue, prior to any processing that may damage the tissue. The collagen-like signal is strong in most meningioma tumors, slightly weaker in glioblastomas, and barely discernable in normal cortex. Further genetic studies would be required to gain insight into which triple helix genes are overexpressed in particular pieces of tissue.

Tumor cells are known for creating unique microenvironments to aid with invasion and proliferation [18]. While further studies involving purification and analysis of cellular and ECM fractions from tissue would be required to determine the molecular specificity of these bands, it seems reasonable to conclude that the infrared signal of the tumors examined thus far have stronger bands in the triple helix region than normal brain tissue. This could be due to the fact that tumor tissue is simply more dense than normal tissue, and thus has a stronger IR signal. Furthermore, brain tumors may have a higher proportion of triple helix molecules in their ECMs than normal tissue [16]. Further investigations on the purified ECMs from tumor tissues and cells will help to determine the contributions of these biomolecules to these bands in the infrared.

## Materials and Methods

### Ethics

All animal studies and protocols were approved by the Regierungspräsidium Dresden (animal ethics committee decision number 24D-9168.11-1/2011-39). All patients gave written consent, and the study was approved by the ethics committee at Dresden University Hospital (decision numbers EK 323122008 and EK 45032004).

### Instrumentation

All measurements were performed on a portable Bruker Alpha-P ATR spectrometer (see figure 1 for photographs of the instrument) using 128 scans and an instrument resolution of 4 cm^−1^. The total measurement time was approximately one minute. This spectrometer is equipped with a diamond crystal (n = 2.43, 0.5 mm^2^). It has an air cooled IR source and an 850 nm HeNe laser. Additionally, it contains a RockSolid interferometer, a deuterated triglycine sulfate detector, and KBr beamsplitters. The angle of incidence is 45°. This instrument is also equipped with an adjustable pressure anvil that aids in forcing as much contact between the crystal and the sample as possible (see 1B, left, arrow). All background measurements were taken with nothing on the crystal. After measurement, the crystal was cleaned first with ethanol, then distilled water, and then with ethanol again.

The collagen thin film was prepared from collagen I (Sigma Aldrich). Its spectrum was acquired with the instrument arm down to enhance the ATR signal.

### ATR measurements of living cells

Primary human gliobastoma cell lines NCH644, NCH421K (kindly provided by Prof. Christel Herold-Mende, Heidelberg) [36], GBM15+X (kindly provided by Prof. Till Acker, Giessen) [37], and murine neuronal stem cells (NSC/C573C6J, kindly provided by Prof. Gaetano Finocchiaro, Milano) [38] were cultivated as floating neurospheres in supplemented DMEM/F-12 medium (PAA). All media contained basic fibroblast growth factor and epidermal growth factor (20 ng/mL each, Peprotech). Cultures were maintained at 37°C in a humidified atmosphere containing 5% CO_2_ and 95%, as previously described [11].

A rat retinal cell culture (R28), a kind gift from Dr. Gail M. Seigel (University at Buffalo, New York) [39], was measured with IR as well. The cells were cultured with T25 flakes in Dulbecco's Modified Eagle Medium (DMEM, Invitrogen) containing: 10% FCS (fetal calf serum, PAN Biotech GmbH), 1% non amino acids (Biochrom AG), 1% MEM vitamin (Biochrom AG), 2% L-glutamine (Biochrom AG), and 1% Na-pyruvate (Biochrom AG), as previously described [39]. For all infrared measurements the adherent cells were trypsinized (trypsin and EDTA solution). The cells were then pelleted and resuspended in pH 7.5 PBS buffer.

To obtain a concentrated single cell suspension, cultured spheroids were titrated with a 10 mL serologic pipette and centrifuged for five minutes at 300 g. The supernatant was removed, and cells were resuspended in a final volume of 50 µl of cell culture medium. To ensure mixing of the sample so a homogenous population of cells were measured, the cells were aspirated 3 to 4 times with a 50 µl pipettman (see figure 1A, right, for a photograph of a cell suspension), and then a 10 µl aliquot was placed on the ATR crystal (see figure 1B, right, for a photograph of the Bruker ATR crystal). This was covered with a CaF^2^ cover slip (see figure 1C, right), and the arm of the instrument was lowered to apply pressure and achieve good contact with the crystal (see figure 1D, right). Cell counts were performed on the cell suspensions to determine the number of cells in a 10 µL aliquot, and this was found to usually be about 10^6^ cells.

### ATR measurements of animal brain tissue

After cervical dislocation of nude mice (NMRI^nu/nu^), the brain was extracted within three minutes of death (see figure 1A, left, boxed). The brain was divided into pieces ranging in weight from 0.05 to 0.07 g (see figure 1A, left, circled). Pieces were placed on the ATR crystal (see figure 1B, left), and the anvil was lowered directly on the the tissue (see figure 1C, left). Adjacent pieces of cortex were saved for control pathology analysis, as well as the measured tissue piece.

### Animal pathology

The pieces of murine brain tissue were embedded in tissue freezing medium (Leica, Nussloch, Germany) and snap frozen on dry ice. Subsequently, sections of 10 µm thickness were prepared with a microcryotome. These sections were then fixed in methanol-acetone (1∶1) and stained with hematoxylin and eosin. The sections were next washed in distilled water, and incubated in Meyer's hematoxylin/hemalaun (Sigma-Aldrich, München, Germany) for three minutes. After washing in distilled water, the tissue sections were briefly destained in HCl-ethanol. The sections were next washed for five minutes in running water, followed by a three minute staining in eosin (1% eosin G in 80% ethanol). The sections were dehydrated with rising ethanol concentrations and cleared in xylene. A coverslip was then placed on the slides using DePex (Serva, Heidelberg, Germany). Images were acquired on a Zeiss Axio Examiner equipped with an Axiocam and 20X objective.

### Patient pathology

After measuring, tissues were immersed in 4% neutrally buffered formalin for at least 24 hours then dehydrated through a series of ethanol baths with increasing concentration (40–100%), immersed in xylene, and then embedded in paraffin. Sections were cut between 3 to 4 µms using a rotational microtome (Leica). The sections were then stained with H&E (Hematoxylin and Eosin, Merck), dehydrated, and mounted. The tumors were visualized and photographed using a Zeiss Axioplan II Microscope. Diagnosis and grading followed according to the WHO classification 2007 at magnifications from 10–40 fold (original) and with the aid of appropriate immunohistochemistry (images available on request) as per Louis 2007 [40].

### Spectral analysis

#### Description of the algorithm

Description of the algorithm used for pre-processing the data. The algorithm was implemented in R [41].

1. Specify endpoints of the region of interest. These should be governed by the specific peak that is of primary interest to researchers.
2. Determine the available wavenumbers closest to the boundary points specified in Step 1, then expand by one to make ensure that the region entirely encompasses the region of interest. The portable IR spectrometer (Bruker) used in this study recorded wavenumbers to one decimal place, therefore some matching is required.
3. Truncate the spectra so that only the region of interest is used in the analysis: this is done for reasons of computational efficiency and ease.
4. Then, for each of the spectra:
  - Create a straight line between the two endpoints of the region of interest. This forms a baseline representing the true absorbtion value of the signal over the instrument's signal to noise ratio.
  - Determine the height (above or below) this baseline and record it. This has the effect of setting the values of the endpoints to zero, and corrects for offsets in the beam path that can occur between identical measurements.
  - If the minimum is below zero after this correction, apply the second stage correction as described in steps d–g.
  - Identify the minimum point: this is done by first finding the minimum value in the range, then recording all values equalling the minimum, and reporting the first/last of those as being the location of the minimum (the options that created the line with the steepest gradient).
  - Create two new baselines between the endpoints and the minimum point(s). The baselines are straight lines from the edge of the region of interest to the minimum point (that is the minimum after the initial baseline correction).
  - Determine the height of each point above the relevant baseline -- for the appropriate part of the spectrum.
  - These correct spectra may now be used to calculate the values for the band heights.

#### Smoothing the curve

In order to smooth the corrected values, a moving average process of length seven was applied for the entire region of interest.

#### Finding the band height and location

When analyzing the 1342 to 1334 cm^−1^ region and the 1325 to 1288 cm^−1^ region the local maxima and minima (height and location) were determined by an internal R [41] function.

## Supporting Information

File S1 **Tables S1, S2, S3, and S4. Table S1 shows the band heights, standard deviations, and number of measurements for two different tissue cell lineages in suspension and the five normal mouse brain tissue pieces used to calculate the average band heights shown in** **table 2** **.** Table S2 shows the raw band heights and positions of the proline and amide III bands for the five normal mice. Table S3 show the raw values calculated for the proline band for the human epilepsy tissue, human meningioma tissue, and human glioblastoma tissue. Table S4 shows the raw calculated values for the amide III bands for the human epilepsy tissue, human meningioma tissue, and human glioblastoma tissue. The values shown in tables S3 and S4 are plotted in figure 10.
(PDF)
