# Supplementary material for: Infrared Spectroscopic Studies of Cells and Tissues: Triple Helix Proteins as a Potential Biomarker for Tumors
Source: PLoS One. 2013 Mar 20;8(3):e58332. doi: 10.1371/journal.pone.0058332 (PMC3604012; doi:10.1371/journal.pone.0058332)
Supplement: File S1 — Tables S1, S2, S3, and S4. Table S1 shows the band heights, standard deviations, and number of measurements for two different tissue cell lineages in suspension and the five normal mouse brain tissue pieces used to calculate the average band heights shown in table 2 . Table S2 shows the raw band heights and positions of the proline and amide III bands for the five normal mice. Table S3 show the raw values calculated for the proline band for the human epilepsy tissue, human meningioma tissue, and human glioblastoma tissue. Table S4 shows the raw calculated values for the amide III bands for the human epilepsy tissue, human meningioma tissue, and human glioblastoma tissue. The values shown in tables S3 and S4 are plotted in figure 10. (PDF) [file pone.0058332.s001.pdf]

Supporting Information File

Table 1: **Height of the amide III band for different cell lineages and animals.**

Sample Type	Height $\pm$ Standard Deviation (AUs)	N
retinal cells passage 1	$0.00045371 \pm 8.65426E-05$	2
retinal cells passage 2	$0.00070603 \pm 5.73205E-06$	2
retinal cells passage 3	$0.00087852 \pm 4.43204E-05$	2
retinal cells passage 4	$0.00071208 \pm 6.29402E-05$	2
tumor cells day 1	$0.00103113 \pm 0.000319376$	2
tumor cells day 2	$0.00092636 \pm 0.000240231$	5
tumor cells day 3	$0.00104668 \pm 0.000209284$	5
animal 1	$0.002677464 \pm 7.554868E-05$	3
animal 2	$0.002398374 \pm 7.756551E-05$	3
animal 3	$0.002195678 \pm 0.0001513385$	3
animal 4	$0.003288499 \pm 0.0005486850$	3
animal 5	$0.002362331 \pm 9.360474E-05$	3

The raw values used to calculate the numbers in table Table 2 are shown with number of measurements (N) that could be performed per experiment for the retinal and tumor cell lineages, as well as the mouse brain tissue.

Table 2: **Height and positions of the 1340 cm^{-1} proline and 1308 cm^{-1} amide III band over five mice.**

Animal and Spectra Number	Height (AUs)	Location (cm^{-1})
animal 1 spectra 1	6.16E-04	1338
animal 1 spectra 2	8.03E-04	1340
animal 1 spectra 3	9.67E-04	1340
animal 2 spectra 1	9.50E-05	1338
animal 2 spectra 2	5.28E-05	1341
animal 2 spectra 3	5.31E-05	1334
animal 3 spectra 1	3.01E-04	1337
animal 3 spectra 2	6.50E-04	1340
animal 3 spectra 3	7.21E-04	1340
animal 4 spectra 1	6.21E-04	1340
animal 4 spectra 2	3.57E-04	1340
animal 4 spectra 3	2.73E-04	1341
animal 5 spectra 1	-3.85E-05	1334
animal 5 spectra 2	-6.73E-05	1334
animal 5 spectra 3	1.91E-05	1334
animal 1 spectra 1	2.60E-03	1310
animal 1 spectra 2	2.74E-03	1310
animal 1 spectra 3	2.69E-03	1311
animal 2 spectra 1	2.48E-03	1308
animal 2 spectra 2	2.38E-03	1308
animal 2 spectra 3	2.33E-03	1308
animal 3 spectra 1	2.36E-03	1310
animal 3 spectra 2	2.17E-03	1308
animal 3 spectra 3	2.06E-03	1310
animal 4 spectra 1	2.66E-03	1313
animal 4 spectra 2	3.60E-03	1310
animal 4 spectra 3	3.61E-03	1308
animal 5 spectra 1	2.26E-03	1308
animal 5 spectra 2	2.37E-03	1308
animal 5 spectra 3	2.45E-03	1308

In normal animal brain tissue, the 1340 cm^{-1} proline band is either very weak or not present. Negative values in this band indicate the band is below the signal to noise of the instrument for wet tissue. The amide III band is centered at 1308 cm^{-1} and has a height of about 0.002 AUs for five animals. In contrast, the proline signal is barely present, if at all.

Table 3: **Calculated values for the heights and positions of the proline band for two patients with epilepsy and patients with meningiomas (n = 10) and glioblastoma multiform (GMB, n = 6).**

Diagnosis	Band height (AUs)	Band position (cm^{-1})
Epilepsy Gray Matter 1	1.72E-04	1341
Epilepsy White Matter 1	6.35E-06	1337
Epilepsy Gray Matter 2	3.63E-04	1338
Epilepsy White Matter 2	1.68E-04	1337
Anaplastic Meningioma WHO III	-8.79E-05	1334
Anaplastic Meningioma WHO III	1.88E-03	1340
Atypical Meningioma WHO II	7.07E-04	1340
Atypical Meningioma WHO II	6.86E-03	1338
Atypical Meningioma WHO II	5.20E-04	1340
Fibrous Meningioma WHO I	1.15E-03	1334
Meningothelial Meningioma WHO I	7.82E-03	1338
Meningothelial Meningioma WHO I	3.40E-03	1338
Meningothelial Meningioma WHO I	9.68E-03	1340
Transitional Meningioma WHO I	2.96E-03	1340
GBM WHO IV	5.73E-04	1338
GBM WHO IV	4.59E-04	1340
GBM WHO IV	6.73E-05	1340
GBM WHO IV	3.81E-03	1338
GBM WHO IV	1.90E-04	1340
GMB WHO IV	7.11E-04	1340

Meningiomas excrete unique ECM molecules, and this is reflected in their infrared spectra as the proline band increases in band height. Glioblastomas have a similar, though much weaker, proline IR signal.

Table 4: **Height and positions of the amide III band for two patients with epilepsy and patients with meningiomas (n = 10) and glioblastoma multiform (GMB, n = 6).**

Diagnosis	Band height (AUs)	Band position(cm^{-1})
Epilepsy Gray Matter 1	1.67E-03	1308
Epilepsy White Matter 1	2.06E-03	1307
Epilepsy Gray Matter 2	3.14E-03	1311
Epilepsy White Matter 2	1.20E-03	1310
Anaplastic Meningioma WHO III	2.32E-03	1304
Anaplastic Meningioma WHO III	2.31E-03	1315
Atypical Meningioma WHO II	1.23E-03	1315
Atypical Meningioma WHO II	3.59E-03	1318
Atypical Meningioma WHO II	8.05E-04	1312
Fibrous Meningioma WHO I	4.90E-03	1306
Meningothelial Meningioma WHO I	3.78E-03	1318
Meningothelial Meningioma WHO I	1.94E-03	1317
Meningothelial Meningioma WHO I	4.55E-03	1318
Transitional Meningioma WHO I	2.22E-03	1315
GBM WHO IV	1.67E-03	1315
GBM WHO IV	1.71E-03	1314
GBM WHO IV	1.91E-03	1300
GBM WHO IV	2.88E-03	1317
GBM WHO IV	1.01E-03	1307
GBM WHO IV	1.03E-03	1317

The amide III vibrations appears to shift in position, a difference that is fairly pronounced in most of the meningiomas and exists to a lesser extent in the glioblastomas. As this band likely has more contributions to its height than the 1340 cm^{-1} proline band, higher variations between individuals with the same diagnosis are expected.
